# Immediate skin-to-skin contact at very preterm birth and effect on infant socio-emotional stress response and mother–infant cortisol co-regulation: secondary outcomes from the randomised clinical Immediate Parent-Infant Skin-To-Skin Study (IPISTOSS)

**DOI:** 10.1136/bmjpo-2025-003778

**Published:** 2026-02-11

**Authors:** Karoline Lode-Kolz, Siri Lillieskold, Hanne Brit Hetland, Eleonora Mascheroni, Stina Klemming, Hanne Pike, Agnes Linner, Nils J Bergman, Bjorn Westrup, Ulrika Ådén, Evalotte Morelius, Rosario Montirosso, Wibke Jonas, Siren Rettedal

**Affiliations:** 1 Department of Clinical Neurophysiology and Department of Paediatrics, Stavanger University Hospital, Stavanger, Norway; 2 Faculty of Health Sciences, University of Stavanger, Stavanger, Norway; 3 Department of Women’s and Children’s Health, Karolinska Institutet, Stockholm, Sweden; 4 Department of Neonatology, Astrid Lindgren Children’s Hospital, Stockholm, Sweden; 5 Department of Biostatistics, Stavanger University Hospital, Stavanger, Norway; 6 0–3 Center for the at-Risk Infant, IRCCS Eugenio Medea, Bosisio Parini, Italy; 7 Lund-Malmö NIDCAP Training and Research Center, Department of Neonatology, Skanes universitetssjukhus Lund, Lund, Sweden; 8 Department of Pediatrics, Stavanger University Hospital, Stavanger, Norway; 9 Department of Clinical Science, Intervention and Technology, Karolinska Institute, Stockholm, Sweden; 10 Department of Health, Medicine and Caring Sciences, Linköping University, Linkoping, Sweden; 11 School of Nursing and Midwifery, Edith Cowan University, Joondalup, Western Australia, Australia; 12 Department of Simulation-based Learning, Stavanger University Hospital, Stavanger, Norway

**Keywords:** Neonatology, Intensive Care Units, Neonatal, Psychology, Endocrinology

## Abstract

**Objective:**

To explore the effects of immediate parent–infant skin-to-skin contact (iSSC) at very preterm birth on maternal–infant behavioural responses and hypothalamic–pituitary–adrenal (HPA) axis reactivity to age-appropriate socio-emotional stress at 4 months corrected age.

**Design:**

Two secondary outcomes from a multicentre randomised controlled trial with two non-blinded parallel groups.

**Setting:**

Three Scandinavian neonatal intensive care units, level 2 and level 3.

**Participants:**

91 infants born at gestational age 28+0 to 32+6 from April 2018 to June 2021. Singletons and twins with a second caregiver present were included, regardless of mode of birth. Higher-order births, infants with congenital infections and major malformations were excluded.

**Intervention:**

Infants were randomised before birth to iSSC (n=46) or conventional care (n=45) during the first 6 hours of life. At 4 months corrected age, the infants were exposed to socio-emotional stress using the Face-to-Face Still-Face procedure (FFSF). Salivary cortisol was collected before and after FFSF.

**Main outcome measures:**

Behavioural and hormonal stress responses at 4 months of age.

**Results:**

65 of 91 infants were assessed by FFSF, of which 37 infants had cortisol sampling. The iSSC group demonstrated heightened positive emotionality during FFSF compared with controls (beta 0.74, 95% CI 0.40 to 1.07; p<0.001). Also, the iSSC group showed an association between mother−infant cortisol levels at baseline (r 0.55, 95% CI 0.13 to 0.80; p=0.014) and 30 min post-FFSF (r 0.55, 95% CI 0.05 to 0.83; p=0.035).

**Conclusion:**

Infants who experienced iSSC during the first 6 hours after very preterm birth showed improved infant socio-emotional stress coping and suggested enhanced mother–infant HPA axis synchrony at 4 months.

**WHAT IS ALREADY KNOWN ON THIS TOPIC**

- Immediate skin-to-skin contact (iSSC) at very preterm birth is safe and reduces neonatal morbidity and mortality. Although recommended by the WHO since 2022 in all settings, iSSC is still not an established practice in numerous high-resource hospitals.

**WHAT THIS STUDY ADDS**

- The Immediate-Parent-Infant-Skin-to-Skin Study is one of the few trials on iSSC at very preterm birth in high-resource settings. Our findings suggest that iSSC at preterm birth could improve emotional and hormonal stress regulation at 4 months corrected age, with enhanced mother–infant cortisol co-regulation.

**HOW THIS STUDY MIGHT AFFECT RESEARCH, PRACTICE OR POLICY**

- Our findings support the new WHO guidelines to avoid parental-infant separation and to implement iSSC for very preterm infants in all settings.

## Introduction

 Complications from preterm birth are a major cause of neonatal morbidity and mortality in low- and high-resource settings.^1 2^ Although progress in perinatal care during the last decades has improved survival, preterm infants remain vulnerable to developmental difficulties in child and young adulthood, both psychological^3 4^ and social.^5 6^ Maternal-infant separation for medical care immediately after preterm birth has for decades been unquestioned, though there is increasing evidence that immediate skin-to-skin contact (iSSC) improves survival and reduces adverse outcomes such as neonatal sepsis and hypothermia.^7^,^9^ The WHO, therefore, in 2022 changed the guidelines for all preterm and low birth weight infants and recommended iSSC as the new standard of care.^10^

Preterm birth and parent-infant separation may induce traumatic stress reactions in both parents and infants, with a heightened risk of socio-emotional and behavioural difficulties.^11 12^

iSSC may facilitate parental–infant interactions, bonding and co-regulation, crucial mechanisms of early bio-behaviour synchrony. Mother–infant coregulation is demonstrated by the coupling of affective and behavioural states in a given moment and over time.^13^ Preterm infants have shown reduced levels of self- and other-directed regulatory behaviours when exposed to socio-emotional stress as compared with term-born infants.^14^

Stress-induced cortisol release is regulated through the hypothalamic–pituitary–adrenal (HPA) axis. Full-term born maternal-infant dyads demonstrate a complementary coregulation within the HPA axis, permitting complex dynamic and reciprocal adaptations in stressful and non-stressful situations. Even though preterm-born infants and mothers can show evidence of HPA axis co-regulation, they appear to be less able to adapt when facing stressful exposures and interactive ruptures as compared with full-term infants.^15^

A limited number of trials have been conducted on the effect of iSSC in high-resource settings. The randomised clinical trial (RCT) IPISTOSS (Immediate Parent-Infant Skin-to-Skin Study) demonstrated improved cardiorespiratory and thermal regulation in the first hours of life.^16 17^ Another RCT in preterm infants also confirmed that iSSC was safe and feasible.^18^ With regard to longer-term outcomes, iSSC has also shown enhanced mother–infant interaction and infant positive affect during play at 4 months,^19^ reduced risk of early postpartum maternal depression and impaired bonding at 6 months.^20^

Our objectives were to explore the effect of iSSC at preterm birth on maternal-infant behavioural responses and HPA-axis reactivity to age-appropriate socio-emotional stress at 4 months corrected age.

## Methods

### Study design

IPISTOSS, a multicentre RCT with two non-blinded parallel groups, was conducted from 1 April 2018 to 30 June 2021 in two neonatal intensive care units (NICUs) at Karolinska University Hospital in Sweden and one at Stavanger University Hospital in Norway. A detailed description can be found in the study protocol.^21^ Independently of allocation, infants were cared for following national and European neonatal resuscitation guidelines. The study was reported according to the Consolidated Standards of Reporting Trials guidelines.

### Patient and public involvement

The research protocol was written in 2015, without formal user involvement. However, the protocol was presented and discussed with parents from the prematurity patient organisations, and their comments were taken into consideration. The intervention was piloted using active patient involvement, informing the research agenda.

### Participants

Pregnant women admitted for threatening preterm birth were screened for eligibility, and both parents gave informed written consent to participate in the study. Inborn singletons and twins with a gestational age (GA) of 28+0 to 32+6 weeks were included regardless of mode of birth. Additional inclusion criteria were that the mother, the other parent or a family member was available for iSSC, and the parents could communicate in Norwegian, Swedish or English. Exclusion criteria were congenital infection, higher-order births than twins, major malformations or other conditions deemed by the attending neonatologist to contraindicate participation. Electronic randomisation before birth was performed (Karolinska Trial Alliance, www.randomize.net) with strata for the three sites and for GA 28+0 to 30+6 and GA 31+0 to 32+6 weeks. All co-parents included in the IPISTOSS happened to be fathers and will be referred to as such.

### Intervention

The primary intervention was defined as initiation of skin-to-skin contact (SSC) with a parent as soon as possible after birth, at the discretion of the neonatologist in charge, and continued for as long as possible throughout the first six postnatal hours. The newborn allocated to iSSC was dried, placed naked on the parent’s bare chest and covered with pre­heated textiles. After vaginal birth, the infant was placed in iSSC with the mother. Transfer to the NICU was carried out in SSC with the mother whenever possible or alternatively, the father. Following Caesarean section or other maternal conditions contraindicating iSSC with the mother, iSSC was initiated with the father and then continued with the mother as soon as she was available. For procedures such as placement of central catheters and radiological examinations, SSC was interrupted only for the time of the procedure.

In the conventional care (CC) group, infants were placed and stabilised in a warmer or incubator during the first 6 hours, including during transfer to the NICU. Parents could hand-hold and participate actively in the care of their newborn.

Time in SSC was logged from 15 min and onwards, during the first 6 hours and throughout the first 8 days, with 15 min accuracy.^22^ Details of SSC duration with IQR during the first 6 hours after birth are presented in table 1. Following the first 6 hours, parents in both groups were equally supported to care for their infants in SSC. Fathers provided more SSC in the first 6 hours than the mothers and continued actively providing SSC in the first 3 and 8 days.

**Table 1:** Patient characteristics

	FFSF assessment		Cortisol assessment	
	Conventional care N=33	Immediate skin-to-skin contact N=32	Conventional care N=18	Immediate skin-to-skin contact N=19
Mothers’ age, years, median (IQR)	32.5 (29.3–34.8)	33.0 (29.0–35.0)	33.0 (28.0–36.0)	34.0 (30.8–35.0)
Two cohabitant parents, n (%)	21 (95)	24 (96)	18 (100)	18 (95)
Education mother, high school, n (%)	3 (14)	7 (28)	3 (17)	4 (21)
Education mother, university, n (%)	19 (86)	15 (60)	15 (83)	12 (63)
Mothers’ prenatal mental health issues, n (%)	2 (9)	4 (17)	2 (11)	4 (21)
Pre-eclampsia, n (%)	7 (32)	8 (32)	6 (33)	5 (26)
Born by caesarean section, n (%)	17 (77)	14 (56)	14 (78)	9 (47)
Twin, n (%)	17 (55)	11 (37)	6 (33)	5 (26)
Sex, male, n (%)	14 (45)	23 (77)	8 (44)	14 (74)
Apgar score at 5 min, median (IQR)	9 (8–9)	9 (8–10)	9 (9–10)	9 (7–10)
Birth weight, g, mean (SD)	1337 (343)	1542 (448)	1362 (407)	1587 (440)
GA total days, median (IQR)	220 (206–224)	223 (215–226)	212 (203–221)	224 (214–226)
GA 28–31 group, n (%)	15 (48)	9 (30)	12 (67)	7 (37)
Accumulated time SSC mother h0–h6, hours, median (IQR)	0.0 (0.0–0.0)	0.6 (0.0–2.8)	0.0 (0.0–0.0)	0.5 (0.0–3.1)
Accumulated time SSC father h0–h6, hours, median (IQR)	0.0 (0.0–0.0)	3.4 (2.3–4.8)	0.0 (0.0–0.0)	3.5 (2.3–5.0)
Total time SSC for infant h0–h6, hours, median (IQR)	0.0 (0.0–0.0)	5.0 (4.4–5.5)	0.0 (0.0–0.0)	5.5 (4.5–5.6)
Accumulated time SSC mother h7–h72, hours, median (IQR)	5.3 (3.5–9.8)	10.6 (6.6–17.3)	5.4 (3.7–9.8)	10.8 (6.6–17.3)
Accumulated time SSC father h7–h72, hours, median (IQR)	3.0 (0.0–4.4)	8.6 (1.9–11.3)	2.7 (0.0–4.6)	8.5 (2.4–11.0)
Total time SSC for infant h7–h72, hours, median (IQR)	9.0 (5.1–13.8)	19.3 (10.7–26.2)	8.9 (4.9–13.4)	19.0 (12.6–25.6)
Accumulated time SSC mother h7–day7, hours, median (IQR)	19.25 (15.5–28.8)	30.5 (21.2–43.6)	17.7 (14.4–27.1)	29.8 (25.1–53.8)
Accumulated time in SSC father h7–day7, hours, median (IQR)	12.3 (6.60–17.20)	24.0 (12.5–30.0)	11.8 (6.8–18.4)	22.4 (17.9–28.0)
Total time SSC for infant h7–day7, hours, median (IQR)	36.5 (24.5–42.5)	53.0 (36.9–70.9)	26.7 (21.5–47.9)	53.5 (36.9–74.4)

N denotes the total number of infants; n denotes the number of infants by item.

FFSF, Face-to-Face Still-Face; GA, gestational age; SSC, skin-to-skin contact.

### Procedures

We report on secondary outcomes collected at 4 months corrected age when the infants had a follow-up visit, including the Face-to-Face Still-Face (FFSF) procedure^23^ and cortisol saliva sampling. The visit was conducted in the morning, primarily at the hospital, though due to the COVID-19 pandemic, some visits were conducted in participants’ homes. During the FFSF procedure, the mother-infant dyad was seated face-to-face with a camera placed to capture their facial expressions. The single-exposure FFSF consists of three episodes called Play1, Still-Face1 and Reunion1, each episode lasting 2 min. During Play1, the mother was to behave as usual when interacting with the infant without using toys or pacifiers. During Still-Face1, she was to look at her infant with a neutral facial expression, not responding in any way when the infant tried to get her attention. In Reunion1, she could interact as usual with the infant. The dyads tested in their homes had the single-exposure FFSF and no cortisol sampling, due to COVID-19 restrictions. The dyads recorded at the hospital had the double-exposure FFSF, meaning that after the three episodes, they followed a second Still-Face2 and Reunion2 episode, and cortisol sampling in mother and infant. The double-exposure FFSF induces a stronger effect on the HPA axis and is preferred for analyses of salivary cortisol post-FFSF.^24^ If a mother came to follow-up with both twins, only the first twin tested with FFSF in the morning had cortisol analysis, as successive FFSF procedures could alter mothers’ cortisol levels. In addition, cortisol has a circadian regulation, and the time of sampling was important.

The FFSF procedure was video recorded, uploaded into ELAN V.6.4 software and time-accurate transcriptions were made of maternal and infant verbal interactions. Coding was performed by three research psychologists, trained in the Global Rating Scales (GRS) of the mother–infant interaction coding system, blinded to the intervention.^25^ Infant behaviours were coded for the first three episodes of FFSF (Play1, Still-Face1, Reunion1), while maternal behaviours were coded for Play1 and Reunion1. Maternal behaviours describe the mother’s sensitivity, intrusiveness and remoteness. Infant behaviours describe the infant’s level of communication, involvement and positive emotionality.

Maternal and infant salivary cortisol samples were collected simultaneously prior to, and at 15 and 30 min after the double-exposure FFSF procedure. The saliva samples were centrifuged at 3000 RPM for 5 min, frozen and stored at −70°C to −80°C.^26^ Cortisol was analysed with ELISA (Salimetrics, Pennsylvania, USA) at the Karolinska Institute and displayed an inter- and intra-assay coefficient of variation of <15% and <4%, respectively. Samples with identified outliers were systematically checked, diluted, reanalysed and confirmed in the laboratory.

### Outcomes

In this study, we report on two secondary outcomes of IPISTOSS, the effect of iSSC on preterm birth: on maternal and infant behaviours during the FFSF procedure, and on the in-moment coupling of infant and maternal salivary cortisol at baseline, 15 and 30 min post-FFSF procedure. As the CC group was recommended to initiate SSC after the first 6 hours, we also studied the effect of the accumulated time of SSC after birth at 72 hours and 8 days on the above.

### Statistical analysis

#### General

Sample size was calculated according to the primary IPISTOSS outcome on physiological transition during the first 6 hours after birth.^21^ Statistical analyses were performed using IBM SPSS Statistics V.26, Stata/SE V.18.0 and R V.4.3.2. Student’s t-test for continuous variables and χ^2^ test for categorical variables were used to compare background characteristics. Independent t-test and Mann-Whitney U test were used for normally and non-normally distributed data, respectively. Data analyses were carried out according to randomisation group, all models were adjusted for twins and the statistical significance level set to a two-sided p value <0.05. The background characteristics that were significantly different between allocations were adjusted for (infants’ sex, GA).

#### Face-to-Face Still-Face

To examine the Still-Face effect, we computed two delta variables separately for the three infant behaviours (level of communication, involvement and positive emotionality): (a) the difference between scores in Play1 and scores in Still-Face1, Delta Still-Face1/Play1; (b) the difference between scores in Still-Face1 and scores in Reunion1, Delta Reunion1/Still-Face1. Similarly, we computed one delta variable for the three maternal behaviours (level of sensitivity, intrusiveness and remoteness): Delta Reunion1/Play1. We used these delta variables to analyse potential differences in infant and maternal behavioural changes during the FFSF procedure between the iSSC and CC groups, using a linear mixed model, reported as mean differences in delta values with 95% CIs and with p values from Wald tests. P values were subsequently corrected for multiple testing with a Bonferroni corrected alpha level of 0.05/12=0.004.

Preliminary Pearson correlations were computed to examine potential associations between infant behavioural changes during the FFSF and maternal behaviour, analysed separately for each intervention group. Based on the association that remained statistically significant after Benjamini-Hochberg False Discovery Rate (FDR) correction, we subsequently tested a moderation model. A stepwise linear regression model was run with infant behavioural change as the outcome variable, and maternal behaviour, intervention group and their interaction term as predictors. The in-moment coupling between infants’ and mothers’ behaviours and groups was studied using a linear mixed model. The same association was tested with adjustment for accumulated time of SSC at 72 hours and at 8 days. Benjamini-Hochberg FDR correction was used to correct for multiple testing.

#### Cortisol

Preliminary and descriptive analysis of cortisol reactivity was based on raw cortisol levels. Since cortisol levels were not normally distributed, they were thereafter log-transformed for use in statistics. Cortisol reactivity was measured as an absolute delta value. The differences in cortisol reactivity in the groups were explored by linear regression with clustered SE, adjusted for country and infants’ sex. Pearson correlation was used for studying the correlation between infants’ and mothers’ log cortisol levels. 95 % CI were calculated with Fisher’s Z transform. Linear regression was used for further exploring the in-moment coupling between infants’ cortisol level, mothers’ cortisol level and randomisation group at different time-points when adjusting for accumulated time of SSC at 72 hours, or during the first 8 days, as well as the interaction between mothers’ cortisol level and accumulated time of SSC at 72 hours or 8 days.

## Results

**Figure 1:**
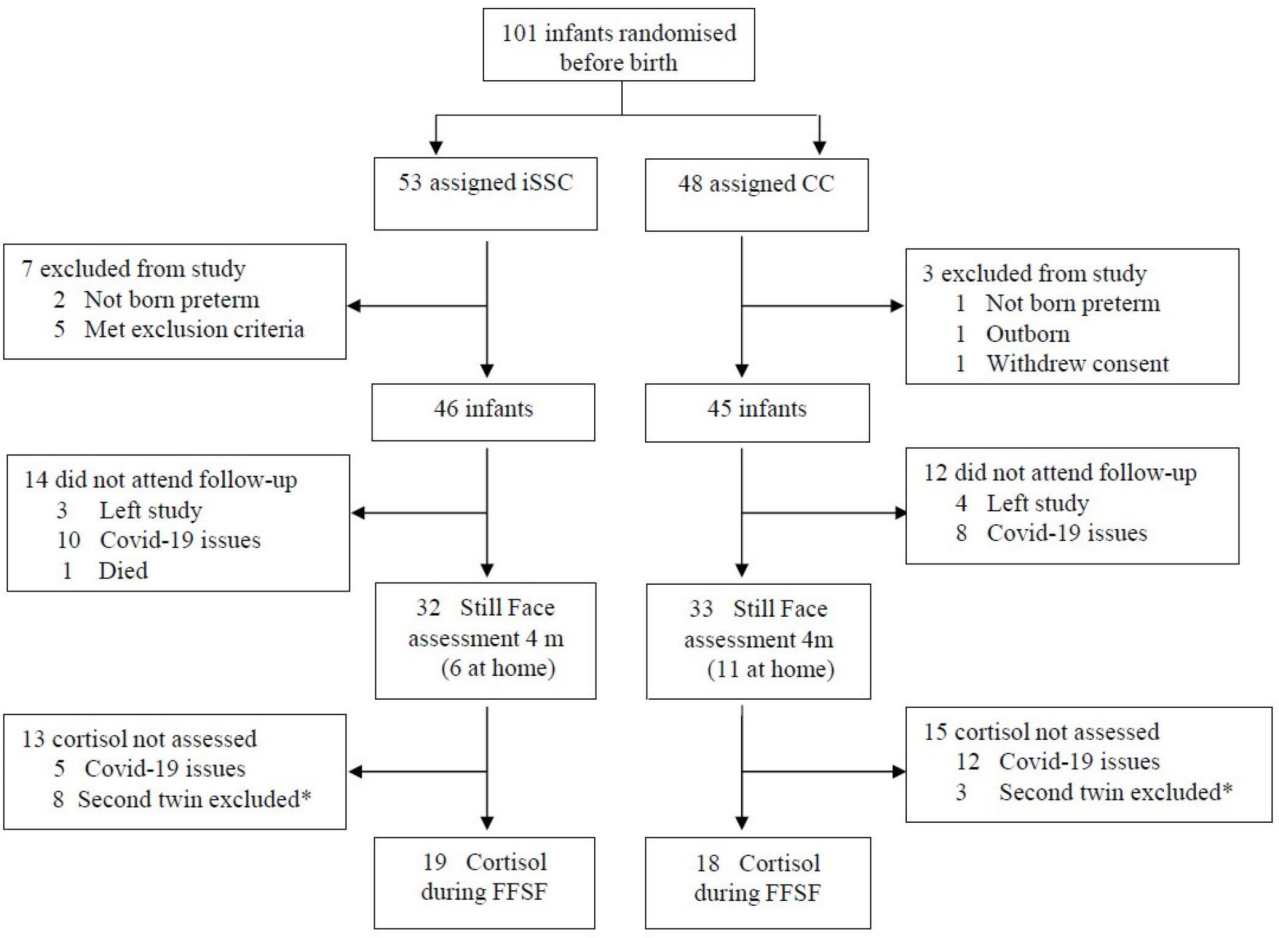
Trial profile. *Only the first mother–twin couplet tested by the FFSF procedure in the morning was included in the cortisol assessment. CC, conventional care; FFSF, Face-to-Face Still-Face; iSSC, immediate skin-to-skin contact.

91 infants were included in the IPISTOSS trial, 46 randomised to iSSC and 45 to CC (figure 1). During the first 6 hours of life, median (IQR) SSC durations in the SSC and CC groups were 5.0 (4.4–5.5) and 0.0 (0.0–0.0) hours, respectively. Separation time during the first 6 hours in the iSSC group was due to the placement of central catheters and radiological examinations. All infants in both groups received Continuous Positive Airway Pressure, except for one from each group that received high-flow treatment. None of the infants was excluded for medical reasons in the first 6 hours.

Due to the COVID-19 pandemic-related attrition, 65 mother-infant dyads were assessed by FFSF, of which 37 had cortisol sampling (figure 1). There were no differences in patient characteristics between the analysed and drop-outs.

Background characteristics were equally distributed for the FFSF paradigm, except for infants’ sex, with a significantly larger proportion of boys in the iSSC group, 23 (77%), as compared with the CC group, 14 (45%) (table 1). In the initial cohort of 91 infants at birth, there was also a significantly larger proportion of boys in the iSSC (72%), as compared with the CC (40%) group. Inter-rater reliability between GRS coders of the FFSF paradigm showed overall scale coefficients (Cohen’s kappa coefficient) between 0.67 and 0.75, with a general agreement of 0.75.^27^ Preliminary analyses of GRS scores were made for country (Norway vs Sweden) and for the observation setting (home vs clinic), with no significant differences in scores between countries or settings.

**Figure 2:**
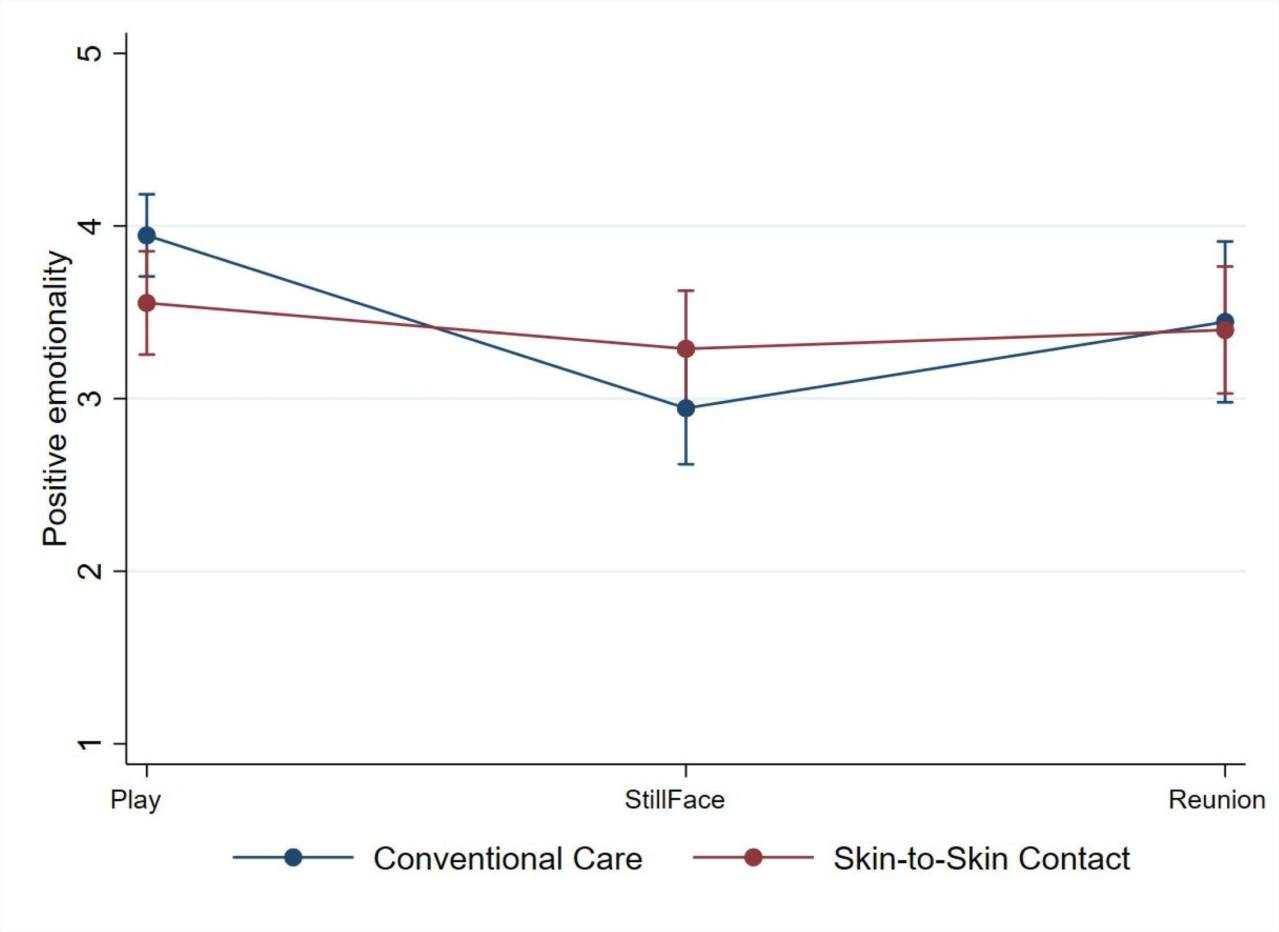
Still-Face effect. Still-Face effect on infants’ positive emotionality. Dots illustrating total Face-to-Face Still-Face scores for infants’ positive emotionality during the three different episodes Play, Still-Face and Reunion. Lines between dots showing the trends in the changes of infants’ positive emotionality from Play to Still-Face and from Still-Face to Reunion.

For infant behaviour, a statistically significant difference was found in infant positive emotionality between the iSSC group and the CC group. Calculated delta variables (delta Still-Face1/Play1 and delta Reunion1/Still-Face1) demonstrated a difference between the iSSC group and the CC group of 0.74 (95% CI 0.40 to 1.07; p<0.001) (figure 2). This remained statistically significant when corrected for multiple comparisons, p<0.004. Univariate analyses for each timepoint did not show differences in infant positive emotionality in Play1, Still-Face1 and Reunion1. No significant results emerged for infant communication and involvement (table 2a).

**Table 2:** FFSF paradigm results

N=65 mother-infant dyads			
(a) Difference in global rating scales scores between the iSSC and CC groups at different timepoints	Beta	95% CI	P value
Infant communication			
Play1	−0.31	−0.68 to 0.07	0.11
Still-Face1	−0.02	−0.41 to 0.37	0.91
Reunion1	0.15	−0.52 to 0.81	0.66
Delta Play1 to Still-Face1	0.29	−0.08 to 0.65	0.13
Delta Still-Face1 to Reunion1	0.17	−0.40 to 0.75	0.56
Infant involvement			
Play1	0.03	−0.26 to 0.33	0.82
Still-Face1	−0.09	−0.44 to 0.27	0.63
Reunion1	−0.08	−0.46 to 0.29	0.66
Delta Play1 to Still-Face1	−0.12	−0.39 to 0.15	0.38
Delta Still-Face1 to Reunion1	0.001	−0.36 to 0.36	0.99
Infant positive emotionality			
Play1	−0.39	−0.80 to 0.02	0.06
Still-Face1	0.34	−0.13 to 0.82	0.16
Reunion1	−0.05	−0.64 to 0.55	0.88
Delta Play1 to Still-Face1	0.74	0.40 to 1.07	<0.001
Delta Still-Face1 to Reunion1	−0.39	−0.88 to 0.10	0.12
Maternal sensitivity			
Play1	−0.08	−0.31 to 0.15	0.48
Reunion1	−0.03	−0.27 to 0.21	0.83
Delta Play1 to Reunion1	−0.11	−0.32 to 0.10	0.31
Maternal intrusiveness			
Play1	0.06	−0.33 to 0.46	0.76
Reunion1	0.24	−0.02 to 0.51	0.08
Delta Play1 to Reunion1	0.18	−0.21 to 0.57	0.36
Maternal remoteness			
Play1	−0.27	−0.71 to 0.18	0.24
Reunion1	−0.09	−0.51 to 0.33	0.67
Delta Play1 to Reunion1	0.18	−0.25 to 0.60	0.42

(b) Difference in positive emotionality between infants in the iSSC and CC groups at different time points mediated by total accumulated time in SSC at 72 hours and 8 days			
Timepoint/mediated by	Beta	95% CI	P value
Delta Play1 to Still-Face1/72 hours	0.75	0.42 to 1.08	<0.001
Delta Still-Face1 to Reunion1/72 hours	−0.32	−0.84 to 0.20	0.23
Delta Play1 to Still-Face1/8 days	0.77	0.44 to 1.09	<0.001
Delta Still-Face1 to Reunion1/8 days	−0.36	−0.88 to 0.16	0.17

N denotes the total number of infants.

CC, conventional care; FFSF, Face-to-Face Still-Face; iSSC, immediate skin-to-skin contact.

Additional analyses on infants’ positive emotionality adjusted for accumulated SSC time at 72 hours or at 8 days showed persistent statistically significant difference in means in favour of the iSSC intervention during the first 6 hours 0.75 (95% CI 0.42 to 1.08; p<0.001) and 0.77 (95% CI 0.44 to 1.09; p<0.001), respectively (table 2b).

Univariate analyses for maternal behaviour at each timepoint did not show differences in Play1 or Reunion1, nor in delta Play1/Reunion1 (table 2a).

**Figure 3:**
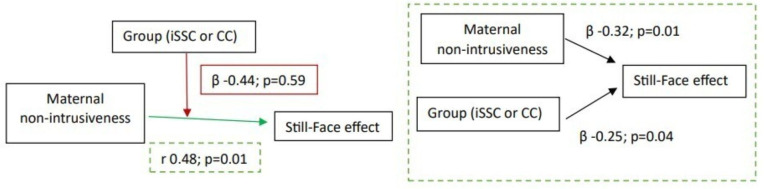
Moderation effect. Both iSSC and mothers’ intrusiveness had an independent effect on the Still-Face effect/positive emotionality in the iSSC group. No moderation effect emerged. To the left: Interaction between maternal non-intrusiveness during Play episode and infant’s positive emotionality in response to Still-Face episode, the Still-Face effect, considering the contribution of iSSC care. To the right: Independent predictors of the Still-Face effect on infants’ positive emotionality. CC, conventional care; iSSC, immediate skin-to-skin contact.

Moreover, we examined correlations between infants’ behaviour in response to the Still-Face paradigm and maternal behaviour during the Play episode, restricting the tests to the only infant behavioural measure that remained significant in the previous analyses (positive emotionality ΔStill-Face1/Play1). Only one association between maternal non-intrusiveness and infant positive emotionality in the iSSC group was statistically significant and survived Benjamini-Hochberg FDR correction. Building on this preliminary finding, we next tested a moderation model. A positive association was confirmed between maternal non-intrusiveness during the Play1 episode and infant’s positive emotionality during Still-Face1 episode only in the iSSC group (r 0.48, 95% CI 0.14 to 0.72; p=0.009). No interaction was found between maternal non-intrusiveness and intervention group (beta −0.44, 95% CI −0.67 to 0.38; p=0.59). Thus, both iSSC and maternal non-intrusiveness independently predicted the Still-Face effect on infants’ positive emotionality (beta −0.32, 95% CI −0.82 to −0.10; p=0.01 and beta −0.25, 95% CI −0.49 to −0.01; p=0.04, respectively) (figure 3).

Background characteristics for cortisol sampling were equally distributed, except a significantly higher GA total days median (IQR) in the SSC group 224 (214.0–226.0) as compared with the CC group 212 (203.0–220.5), p=0.003 (table 1).

A preliminary analysis of mother and infant cortisol levels was made for each country at baseline, 15 and 30 min post-FFSF. No significant difference emerged.

Descriptive analysis of cortisol levels in the iSSC and the CC groups did not show significant differences (table 3a). There were no significant differences in delta cortisol between iSSC and CC groups from baseline to 15 min or 30 min post FFSF procedure (table 3b). We analysed the in-moment cortisol coupling at each time point (ie, baseline, 15 min and 30 min), separately for the two groups (iSSC and CC). There was a significant association between infants’ and mothers’ cortisol levels for the iSSC group at baseline (r 0.55, 95% CI 0.13 to 0.80; p=0.014), not found in the CC group (r 0.11, 95% CI −0.38 to 0.55; p=0.67). No association was found between infants’ and mothers’ cortisol levels in the iSSC and CC groups at 15 min. At 30 min post-FFSF, there was a significant association between infants’ and mothers’ cortisol levels for the iSSC group (r 0.55, 95% CI 0.05 to 0.83; p=0.035), not present in the CC group (r 0.28, 95% CI −0.23 to 0.67; p=0.27) (table 3c).

**Table 3:** Cortisol results

N=37 mother-infant dyads			
(a) Raw cortisol levels (nmol/L)			
Timepoint	Immediate skin-to-skin contact N=19	Conventional care N=18	P value
Baseline infant, median (IQR), range, n=37	6.99 (3.71–16.96) 2.31–187.00	7.10 (4.71–10.14) 1.86–14.41	0.48
15 min infant, median (IQR), range, n=34	8.18 (4.65–16.90) 1.89–544.36	6.14 (3.94–11.66) 3.37–20.23	0.50
30 min infant, median (IQR), range, n=32	6.62 (3.62–17.16) 1.59–121.47	5.15 (3.77–12.23) 2.46–24.88	0.43
Baseline mother, median (IQR), range, n=37	5.54 (4.27–10.51) 2.07–35.72	6.35 (4.99–11.63) 3.91–18.66	0.94
15 min mother, median (IQR), range, n=35	3.47 (2.64–7.81) 1.35–76.62	5.77 (3.47–7.73) 1.11–12.62	0.81
30 min mother, median (IQR), range, n=32	4.17 (1.81–5.79) 1.10–55.34	4.93 (2.74–7.35) 1.23–12.12	0.71

(b) Differences between iSSC and CC groups in cortisol reactivity from baseline to 15 and 30 min post Still-Face assessment			
Delta log-transformed cortisol levels	Beta	95 % CI	P value
Baseline−15 min infant, n=34	11.44	−8.77 to 31.65	0.26
Baseline−30 min infant, n=32	7.43	−1.93 to 16.78	0.12
Baseline−15 min mother, n=35	0.58	−3.08 to 4.23	0.75
Baseline−30 min mother, n=32	−0.28	−2.86 to 2.30	0.83

(c) Association between infants’ and mothers’ log-transformed cortisol levels at different time points, in iSSC and CC groups			
Timepoint	r	95 % CI	P value
CC baseline, n=18	0.11	−0.38 to 0.55	0.67
CC 15 min, n=17	0.43	−0.01 to 0.75	0.09
CC 30 min, n=17	0.28	−0.23 to 0.67	0.27
iSSC baseline, n=19	0.55	0.13 to 0.80	0.014
iSSC 15 min, n=17	0.42	−0.07 to 0.75	0.09
iSSC 30 min, n=15	0.55	0.05 to 0.83	0.035

N denotes the total number of infants; n denotes the number of infants by item.

CC, conventional care; iSSC, immediate skin-to-skin contact.

Additional analyses on the association between infants’ and mothers’ cortisol levels adjusted for total accumulated SSC time at 72 hours or at 8 days showed no significant findings.

## Discussion

Our findings suggest that iSSC in the first 6 hours of life improves behavioural and hormonal responses to stress measurable at 4 months corrected age, compared with SSC starting after 6 hours.

CC infants showed decreased positive emotionality during the maternal unresponsiveness episode, while infants in the iSSC group expressed a stable positive emotionality. These findings suggest that SSC during the first hours of life may be associated with enhanced socio-emotional stress coping. Furthermore, our findings of a significant association between maternal intrusiveness and infant positive emotionality in the iSSC group suggest that a sensitive maternal behaviour during the play episode could play a role in supporting infants’ behavioural regulation during the challenging condition provoked by the maternal unresponsiveness episode.^28^

Although exploratory in nature, the analyses of infant and mother’s in-moment coupling salivary cortisol levels revealed two contrasting trends of association between study groups. In the CC group, no significant associations were found at any of the measured time points, indicating an absence of HPA axis coupling. This may suggest that infants and mothers of the CC group face challenges in co-regulating biological rhythms, particularly when confronted with interactive disruptions or stressful situations. In contrast, in the iSSC group, significant associations were observed at baseline and 30 min, with no association at 15 min. This finding seems to suggest that iSSC might promote coupled salivary cortisol levels during a calm state, which is prior to the occurrence of the socio-emotional stress condition. Furthermore, while the coupling was temporarily disrupted at 15 min following socio-emotional stress, a significant coupling between mothers and infants was reached again during recovery at 30 min. We speculate that the iSSC may have facilitated the infant’s ability to manage stress and re-establish a biological homeostatic balance, as in full-term infants^29^ in contrast to the dampened stress reactivity usually described in preterms.^30^ Similar effects of iSSC have recently been described in both late^26^ and extremely preterm infants with reduced expression of stress-related genes and enhanced HPA axis co-regulation.^30^

The iSSC infants received significantly more time in SSC at all time points during the first 8 days compared with the CC group. This is not explained by the number of hours of SSC during the intervention period, nor access to SSC during the first week. Parents in both groups had equal opportunities to provide SSC after the first 6 hours. We hypothesise that this difference in SSC time is an effect of the iSSC intervention, which might have led to heightened awareness, empowerment and care coping skills in the iSSC parents. In this trial, fathers were the main caregivers during the iSSC intervention and interestingly, they provided twice as much SSC time, from 7 hours and during the first week, compared with fathers in the CC group. These findings align with former findings from the IPISTOSS study, where iSSC showed a reduction in symptoms of depression in mothers and anxiety in fathers 1 week after birth.^31^ We can speculate that the effect on parents’ well-being enabled their capacity for parent–infant interaction, in favour of early bonding. Our findings align with previous findings from the IPISTOSS, showing that iSSC had a positive impact on infants’ behaviour with a higher infant positive affect and communicative skills in social situations at 4 months,^19^ and enhanced language skills at 2 years.^32^ Interestingly, the present and previous results from IPISTOSS demonstrated significant benefits of iSSC, although the father provided most of the SSC during the first 6 hours.^16 17 19 32^ Thus, even though mother–infant couplet care with zero separation should be favoured whenever possible, these reports combined highlight the importance of the presence of the other parent in these first hours of life to ensure SSC as soon as possible after birth when the mother is unavailable.^33^

Current practice assumes early separation after preterm birth to be safe. This study shows differences in stress responses at 4 months from an early and short iSSC intervention provided at birth. The concept of a vulnerability window aligns with our hypothesis on a critical period immediately after preterm birth, impacting the early settings of the HPA axis and stress resilience.^34^

The present study has several strengths. The randomised design decreased the risk of a selection bias and assured homogenous study groups, considering site and age groups. Independent of the randomisation group, the infants received the same care; only the place of care differed during the first 6 hours of life. There were very few differences in characteristics between the infants analysed and drop-outs, reducing the risk of selection bias. For cortisol sampling, there was a difference in infants’ GA, with GA total days median (IQR) in the SSC group 224 (214.0–226.0) versus CC group 212 (203.0–220.5), p=0.003. This could have influenced our results, but was corrected for in the statistical analysis. The multisite design and the absence of differences in FFSF score and cortisol levels between sites strengthen the reproducibility of the intervention and quality of the results. Even though the 4-month follow-up was carried out in different settings, no differences in FFSF scores emerged between home or clinic settings. Moreover, there was a high inter-rater reliability reflecting high fidelity of the procedures.

In terms of limitations, the sample size was smaller than planned as the main trial was terminated before reaching the projected sample size due to pandemic-related recruitment restrictions, an issued press release from the WHO promoting a procedure shift to iSSC also in high-resource settings, and significant beneficial effects of the primary outcome on cardiorespiratory stability were demonstrated in a preliminary analysis. The sample size, due to early study termination and attrition during follow-up, may have been a limitation affecting the revelation of minor differences between groups.

## Conclusion

Infants who experienced iSSC during the first 6 hours after very preterm birth showed improved infant positive emotionality as a behavioural response to socio-emotional stress. In addition, our findings suggest an enhanced mother-infant HPA axis coupling at 4 months. These results support the new WHO guidelines to avoid parental-infant separation and to implement iSSC for very preterm infants in all settings.

## Data Availability

When obtaining consent from the parents included in the study, we have not specifically asked for their permission to share data with the larger scientific community, and therefore, data will not be available.

## References

[1] UNICEF. https://data.unicef.org/resources/levels-and-trends-in-child-mortality-2024.

[2] WHO. https://www.who.int/news-room/fact-sheets/detail/newborn-mortality.

[3] Vanes LD, Murray RM, Nosarti C (2022). Adult outcome of preterm birth: Implications for neurodevelopmental theories of psychosis. Schizophr Res.

[4] Bachmann CS, Risnes K, Bjørngaard JH (2021). Association of Preterm Birth With Prescription of Psychotropic Drugs in Adolescence and Young Adulthood. JAMA Netw Open.

[5] Ni Y, Mendonça M, Baumann N (2021). Social Functioning in Adults Born Very Preterm: Individual Participant Meta-analysis. Pediatrics.

[6] Mendonça M, Bilgin A, Wolke D (2019). Association of Preterm Birth and Low Birth Weight With Romantic Partnership, Sexual Intercourse, and Parenthood in Adulthood: A Systematic Review and Meta-analysis. JAMA Netw Open.

[7] Arya S, Naburi H, Kawaza K (2021). Immediate “Kangaroo Mother Care” and Survival of Infants with Low Birth Weight. N Engl J Med.

[8] Worku B, Kassie A (2005). Kangaroo mother care: a randomized controlled trial on effectiveness of early kangaroo mother care for the low birthweight infants in Addis Ababa, Ethiopia. J Trop Pediatr.

[9] Bergman NJ, Linley LL, Fawcus SR (2004). Randomized controlled trial of skin-to-skin contact from birth versus conventional incubator for physiological stabilization in 1200- to 2199-gram newborns. Acta Paediatr.

[10] WHO (2022). WHO recommendations for care of the preterm or low-birth-weight infant.

[11] Bergman NJ (2019). Birth practices: Maternal-neonate separation as a source of toxic stress. Birth Defects Res.

[12] Sanders MR, Hall SL (2018). Trauma-informed care in the newborn intensive care unit: promoting safety, security and connectedness. J Perinatol.

[13] Feldman R (2020). What is resilience: an affiliative neuroscience approach. World Psychiatry.

[14] Provenzi L, Fumagalli M, Bernasconi F (2017). Very Preterm and Full-Term Infants’ Response to Socio-Emotional Stress: The Role of Postnatal Maternal Bonding. Infancy.

[15] Provenzi L, Giusti L, Fumagalli M (2019). The dual nature of hypothalamic-pituitary-adrenal axis regulation in dyads of very preterm infants and their mothers. Psychoneuroendocrinology.

[16] Linnér A, Lode Kolz K, Klemming S (2022). Immediate skin-to-skin contact may have beneficial effects on the cardiorespiratory stabilisation in very preterm infants. Acta Paediatr.

[17] Lode-Kolz K, Hermansson C, Linnér A (2023). Immediate skin-to-skin contact after birth ensures stable thermoregulation in very preterm infants in high-resource settings. Acta Paediatr.

[18] Kristoffersen L, Bergseng H, Engesland H (2023). Skin-to-skin contact in the delivery room for very preterm infants: a randomised clinical trial. BMJ Paediatr Open.

[19] Lilliesköld S, Lode-Kolz K, Rettedal S (2023). Skin-to-Skin Contact at Birth for Very Preterm Infants and Mother-Infant Interaction Quality at 4 Months: A Secondary Analysis of the IPISTOSS Randomized Clinical Trial. JAMA Netw Open.

[20] Mehler K, Hucklenbruch-Rother E, Trautmann-Villalba P (2020). Delivery room skin-to-skin contact for preterm infants-A randomized clinical trial. Acta Paediatr.

[21] Linnér A, Westrup B, Lode-Kolz K (2020). Immediate parent-infant skin-to-skin study (IPISTOSS): study protocol of a randomised controlled trial on very preterm infants cared for in skin-to-skin contact immediately after birth and potential physiological, epigenetic, psychological and neurodevelopmental consequences. BMJ Open.

[22] Axelin A, Raiskila S, Lehtonen L (2020). The Development of Data Collection Tools to Measure Parent-Infant Closeness and Family-Centered Care in NICUs. Worldviews Evid Based Nurs.

[23] Zuckerman B, Tronick E (2023). The Still-Face Paradigm: Training Model for Relational Health. J Dev Behav Pediatr.

[24] Provenzi L, Giusti L, Montirosso R (2016). Do infants exhibit significant cortisol reactivity to the Face-to-Face Still-Face paradigm? A narrative review and meta-analysis. Dev Rev.

[25] Fiori-Cowley A, Murray L, Gunning M (2000). Global ratings of mother–infant interaction at two and four months.

[26] Mörelius E, Örtenstrand A, Theodorsson E (2015). A randomised trial of continuous skin-to-skin contact after preterm birth and the effects on salivary cortisol, parental stress, depression, and breastfeeding. Early Hum Dev.

[27] McHugh ML (2012). Interrater reliability: the kappa statistic. Biochem Med (Zagreb).

[28] Braungart-Rieker JM, Zentall S, Lickenbrock DM (2014). Attachment in the making: mother and father sensitivity and infants’ responses during the Still-Face Paradigm. J Exp Child Psychol.

[29] Provenzi L, Giusti L, Fumagalli M (2016). Pain-related stress in the Neonatal Intensive Care Unit and salivary cortisol reactivity to socio-emotional stress in 3-month-old very preterm infants. Psychoneuroendocrinology.

[30] Hucklenbruch-Rother E, Vohlen C, Mehdiani N (2020). Delivery room skin-to-skin contact in preterm infants affects long-term expression of stress response genes. Psychoneuroendocrinology.

[31] Lilliesköld S, Lode-Kolz K, Westrup B (2025). Skin-to-skin contact at birth for very preterm infants and symptoms of depression and anxiety in parents during the first year - A secondary outcome of a randomized clinical trial. J Affect Disord.

[32] Lode-Kolz K, Jonas W, Hetland HB (2025). Immediate Skin-to-Skin Contact at Very Preterm Birth and Neurodevelopment the First Two Years: Secondary Outcomes from a Randomised Clinical Trial. Children (Basel).

[33] Bakermans-Kranenburg MJ, Lotz A, Alyousefi-van Dijk K (2019). Birth of a Father: Fathering in the First 1,000 Days. Child Dev Perspect.

[34] Feldman R (2015). Sensitive periods in human social development: New insights from research on oxytocin, synchrony, and high-risk parenting. Dev Psychopathol.

