# Supplementary material for: Immediate skin-to-skin contact at very preterm birth and effect on infant socio-emotional stress response and mother–infant cortisol co-regulation: secondary outcomes from the randomised clinical Immediate Parent-Infant Skin-To-Skin Study (IPISTOSS)
Source: BMJ Paediatr Open. 2026 Feb 11;10(1):e003778. doi: 10.1136/bmjpo-2025-003778 (PMC12911832; doi:10.1136/bmjpo-2025-003778)
Supplement: Supplementary file 1 [file bmjpo-2025-003778.reviewer_comments.pdf]

PEER REVIEW HISTORY

BMJ Paediatrics Open publishes all reviews undertaken for accepted manuscripts. Reviewers are asked to complete a checklist review form and are provided with free text boxes to elaborate on their assessment. These free text comments are reproduced below.

ARTICLE DETAILS

**TITLE (PROVISIONAL)**	Immediate skin-to-skin contact at very preterm birth and effect on infant socio-emotional stress response and mother-infant cortisol co-regulation: secondary outcomes from the randomised clinical Immediate Parent-Infant Skin-To-Skin Study (IPISTOSS)
**AUTHORS**	Lode-Kolz, Karoline<br>Lillieskold, Siri<br>Hetland, Hanne Brit<br>Mascheroni, Eleonora<br>Klemming, Stina<br>Pike, Hanne<br>linner, agnes<br>Bergman, Nils J<br>Westrup, Bjorn<br>Ådén, Ulrika<br>Morelius, Evalotte<br>Montirosso, Rosario<br>Jonas, Wibke<br>Rettedal, Siren

VERSION 1 – REVIEW

**REVIEWER NAME**	Niermeyer, Susan
**REVIEWER AFFILIATION**	University of Colorado Denver School of Medicine, Pediatrics
**REVIEWER CONFLICT OF INTEREST**	No Conflict of Interest
**AI DISCLOSURE**	No, I have not used AI
**DATE REVIEW RETURNED**	23-Jul-2025

**GENERAL COMMENTS**	The manuscript describes secondary socio-emotional and stress response outcomes among participants in the IPISTOSS randomized controlled trial of immediate skin-to-skin contact at very preterm birth. The findings add valuable insights on the behavioral and hormonal effects of immediate skin-to-skin care. Significant loss of RCT participants to follow-up during the COVID pandemic limits the strength of the findings. However, the controlled trial in a high-resource setting with robust data collection is very important to establish the benefit of immediate skin-to-skin care in settings where perinatal infection and mortality are already quite low.<br><br>Abstract: The abstract accurately reflects the data presented in the manuscript. The expression of gestational age is somewhat awkward in the format weeks + days. The conclusion could be improved by adding “Infants who experienced” to the sentence.<br><br>What This Study Adds: The statement that “iSSC at preterm birth improves…hormonal stress regulation at 4 months corrected age” may stretch somewhat beyond the data presented.

	Introduction: The introduction is concise and offers useful background on the hormonal aspects of stress response and resilience, but less background on behavioral aspects. As behavioral disorders are such common consequences of prematurity, further background would be useful. The modifying phrase “less developed after preterm birth” (line 39) has an unclear referent.<br><br>Materials and methods: The separate study protocol describes that if stabilization was likely to take more than 5 minutes at birth, the infant might be placed first under a radiant warmer. Intubation was also a reason for interruption of skin-to-skin contact. These factors that reflect the degree of intervention received and possible interruption of skin-to-skin contact deserve mention in the manuscript.<br>Was there physical contact (touch) between mother and newborn infant in the delivery setting for the conventional care group?<br>The methods section might also mention the reason that cortisol sampling was not performed on second twins.<br><br>Results: The results are presented in an organized way. Further details on types of medical intervention required in both groups (and separation time in the first 6 hours) would add further context to the results.<br>It may be incorrect to interpret the p-value for the confidence interval of the beta coefficient as “significance” of the finding. The confidence intervals are very wide, and the p-value tests the null hypothesis that the correlation coefficient is zero.<br>Discussion: The discussion should touch on the implication of observed imbalances in the follow-up cohort (sex differences for FFSF paradigm and gestational age for cortisol testing). Although the original RCT design decreased the risk of imbalances between the control and intervention groups, the loss of trial participants in the follow-up study resulted in imbalances.<br><br>Conclusions: The conclusion reflects the data accurately.<br><br>References: References are pertinent and current.<br><br>Tables and Figures: Figures and tables are clearly presented. The significant imbalances in subject characteristics could be noted on the table.<br><br>Spelling, syntax: Spelling of Bergman, Nils should be corrected on the cover page if this is used for indexing information.<br><br>Page 9, line 25 “recommend” should be “recommended”.

**REVIEWER NAME**	**Dalrymple, Hannah**
**REVIEWER AFFILIATION**	The Children's Hospital at Westmead, Grace NICU
**REVIEWER CONFLICT OF INTEREST**	No Conflict of Interest
**AI DISCLOSURE**	No, I have not used AI
**DATE REVIEW RETURNED**	22-Oct-2025

**GENERAL COMMENTS**	This is an interesting paper that is very well written and explores an important area. It is of course a shame that your results were

	disrupted by the Covid-19 pandemic and I commend you on finding a sensible way to analyse data despite this.<br><br>I note a few areas where I believe the discussion of your results could be enhanced.<br><br>Firstly you have analysed a large number of variables related to the intervention groups and found only a few with statistical significance. How sure can you be that this is an intervention effect or is it just chance? Do you have a postulated theory as to why those particular variables were significant but not other measures of behaviour or stress? The first sentence of the discussion is too categorical for me, I am not sure that your results support the statement.<br><br>I am particularly interested in the fact that the infants in the SSC group received much higher total SSC across all timepoints in the first 7 days compared with the CC group (53 vs 36.5 hours). The difference is not purely explainable by the hours in the first 6 hours in the intervention group. I would like to see you address the difference in total SSC between groups as part of the discussion and analysis as although the first 6 hours may well be a vulnerable period perhaps the emphasis on importance of SSC or early initiation lead to more comfort of parents and thus more SSC throughout the admission in those who commence earlier. Is it that more SSC leads to these changes in cortisol coupling and behavioural response, not just the SSC in the first 6 hours?

VERSION 1 – AUTHOR RESPONSE

In black: reviewers comments/questions.

In blue our answers and In red our changes in the manuscript

Reviewer 1

Abstract:

The expression of gestational age is somewhat awkward in the format weeks + days.

We agree, and have modified this to page 3: 91 infants born at gestational age 28+0 to 32+6 from April 2018 to June 2021

The conclusion could be improved by adding “Infants who experienced” to the sentence.

Thank you for this suggestion, we agree and have modified to page 3: Infants who experienced iSSC during the first six hours after very preterm birth demonstrated improved socio-emotional stress coping and suggests enhanced mother-infant HPA axis synchrony at four months.

What This Study Adds :

iSSC at preterm birth improves…hormonal stress regulation at 4 months corrected age

Thank you for this comment, we have modified to page 4: Our findings suggests that iSSC at preterm birth could improve emotional and hormonal stress regulation at 4 months corrected age, with enhanced mother-infant cortisol co-regulation.

Introduction :

The introduction is concise and offers useful background on the hormonal aspects of stress response and resilience, but less background on behavioral aspects. As behavioral disorders are such common consequences of prematurity, further background would be useful.

Our modifications considering the behavioural aspects of stress have been added page 5:

"Preterm birth and parent-infant separation may induce traumatic stress reactions in both parents and infants, with a heightened risk of socio-emotional and behavioural difficulties.[11,12] iSSC may facilitate parental-infant interactions, bonding, and co-regulation, crucial mechanisms of early bio-behaviour synchrony. Mother-infant coregulation is demonstrated by coupling of affective and behavioural states in a given moment and over time.[13] Preterm infants have shown reduced levels of self- and other directed regulatory behaviours when exposed to socio-emotional stress as compared to term born infants.[14]

Materials and methods:

The separate study protocol describes that if stabilization was likely to take more than 5 minutes at birth, the infant might be placed first under a radiant warmer. Intubation was also a reason for interruption of skin-to-skin contact. These factors that reflect the degree of intervention received and possible interruption of skin-to-skin contact deserve mention in the manuscript.

We did not register if infants were put in iSSC directly and during the first 14 minutes, only from 15minutes onwards. I have added this to the manuscript for more clarity page 8:

"Time in SSC was logged from minutes 15 and onwards, during the first six hours and throughout the eight first days, with 15-minute accuracy." Page 8

We also added this in results Page 12: "Ninety-one infants were included in the IPISTOSS trial, 46 randomised to iSSC and 45 to CC. Figure 1 During the first six hours of life, median (interquartile range, IQR) SSC durations in the SSC and CC groups were 5.0 (4.4, 5.5) and 0.0 (0.0, 0.0) hours, respectively. Separation time during the first 6 hours in the iSSC group was due to placement of central catheters and radiological examinations. All infants in both groups received Continuous Positive Airway Pressure except for one from each group that received high-flow treatment. None of the infants were excluded for medical reasons the first six hours."

Was there physical contact (touch) between mother and newborn infant in the delivery setting for the conventional care group?

Concerning physical contact we have modified from "Parents could remain and participate actively in the care of their newborn" to "Parents could hand-hold and participate actively in the care of their newborn." Page 8

Concerning cortisol and twins we have added: "If a mother came to follow-up with both twins, only the first twin tested with FFSF in the morning had cortisol analysis, as successive FFSF procedures could alter mothers´ cortisol levels. In addition, cortisol has a circadian regulation, and the time of sampling was important." Page 9

Results:

Further details on types of medical intervention required in both groups (and separation time in the first 6 hours) would add further context to the results.

Thank you for this important comment, as described earlier we have added this to results page 11

It may be incorrect to interpret the p-value for the confidence interval of the beta coefficient as “significance” of the finding. The confidence intervals are very wide, and the p-value tests the null hypothesis that the correlation coefficient is zero. We have modified our formulations in the discussion and conclusion in order to appear less affirmative.

Discussion :

Concerning imbalances in sex , this was already present in the initial cohort and was corrected for in our other articles both in primary outcome and in secondary outcomes at four months and two years. I have added in Methods, Statistical analysis, for more clarity page 10: «The background characteristics that were significantly different between allocations were adjusted for (infants´sex and GA)

and a sentence on this in results page 12: «In the initial cohort of 91 infants at birth there was also a significantly larger proportion of boys in the iSSC (72%) as compared to CC (40%) group.» And in discussion page 16: «For cortisol sampling there was a difference in infants´ GA, with GA total days median (IQR) in the SSC group 224 (214.0-226.0) versus CC group 212 (203.0-220.5), p=0.003. This could have influenced our results, but was corrected for in the statistical analysis.»

Spelling of Bergman, Nils is corrected at cover page. Thank you for pointing this out for us.

Conclusion :

I have modified the conclusion as follows page 17:

«Infants who experienced iSSC during the first six hours after very preterm birth showed improved infant positive emotionality as a behavioural response to socio-emotional stress. In addition, our findings suggest an enhanced mother-infant HPA axis coupling at four months. These results support the new WHO guidelines to avoid parental-infant separation and to implement iSSC for very preterm infants in all settings. .»

Reviewer 2

You have analysed a large number of variables related to the intervention groups and found only a few with statistical significance. How sure can you be that this is an intervention effect or is it just chance? Do you have a postulated theory as to why those particular variables were significant but not other measures of behaviour or stress?

We agree that a lot of tests were done, counting all comparisons of delta values for six Still Face outcomes we have 12 tests. Meaning that even with the conservative Bonferroni correction, our one finding will still be considered statistically significant (p<0.004). We have added this information in methods page 10-11 “We used these delta variables to analyse potential differences in infant and maternal behaviour trends during the FFSF procedure between the iSSC and CC groups, using linear mixed model, reported as mean differences in delta values with 95% confidence intervals (CI) and with p-values from Wald tests. P-values were subsequently compared with a Bonferroni corrected alpha level of 0.05/12=0.004” and results page 12: “This remained statistically significant when corrected for multiple comparisons, p< 0.004”.. Even so, there is of course a possibility that the statistically significant finding is due to chance. However, the findings align with findings in the literature, as described in the introduction. We have modified our formulations in the discussion and conclusion in order to appear less affirmative.

In addition, when exploring potential associations between infants' behavior during the Face-to-face Still-Face episodes and maternal behavior during the Play episode, we deliberately limited the number of correlations to reduce the risk of type I error. Specifically, we examined only the infant behavioral variable that remained significant in the previous analyses (positive emotionality ΔStill-Face1/Play1), thereby restricting the number of multiple tests. For these remaining correlations, only one association between maternal non-intrusiveness and infant positive emotionality ΔStill-Face1/Play1 in the iSSC group was significant and survived to the Benjamini–Hochberg False Discovery Rate (FDR) correction. Page 13: "Moreover, we examined correlations between infants' behaviour in response to the Still-Face paradigm and maternal behaviour during the Play episode, restricting the tests to the only infant behavioural measure that remained significant in the previous analyses (positive emotionality ΔStill-Face1/Play1). Only one association between maternal non-intrusiveness and infant positive emotionality in the iSSC group, was statistically significant and survived Benjamini–Hochberg FDR correction. Building on this preliminary finding, we next tested a moderation model. A positive association was confirmed between maternal non-intrusiveness during the Play1 episode and infant's positive emotionality"

Finally, regarding the cortisol analyses, we examined a small set of six pre-planned, theory-driven correlations (three time points × two groups) based on prior evidence on mother–infant HPA axis coupling. Given the limited number of hypothesis-driven comparisons, we reported unadjusted p-values and interpret the cortisol coupling results cautiously, as supportive and consistent with the literature rather than definitive. We have now used more cautious language in the discussion sections of the manuscript, employing less affirmative statements.

I am particularly interested in the fact that the infants in the SSC group received much higher total SSC across all timepoints in the first 7 days compared with the CC group (53 vs 36.5 hours). The difference is not purely explainable by the hours in the first 6 hours in the intervention group. I would like to see you address the difference in total SSC between groups as part of the discussion and analysis as although the first 6 hours may well be a vulnerable period perhaps the emphasis on importance of SSC or early initiation lead to more comfort of parents and thus more SSC throughout the admission in those who commence earlier. Is it that more SSC leads to these changes in cortisol coupling and behavioural response, not just the SSC in the first 6 hours?

The Still Face results (infant positive emotionality) was corrected for time spent in skin-to -skin after the intervention, and still the difference between groups persisted

I have added this in the discussion page 16:

The iSSC infants received significantly more time in SSC at all timepoints during the first eight days compared to the CC group. This is not explained by the number of hours of SSC during the intervention period nor access to SSC during the first week. Parents in both groups had equal opportunities to provide SSC after the six first hours. We hypothesise that this difference in SSC time is an effect of the iSSC intervention, that might have led to heightened awareness, empowerment and care coping skills in the iSSC parents. In this trial, fathers were the main care givers during the iSSC intervention and interestingly they provided twice as much SSC time from seven hours and during the first week compared to fathers in the CC group. These findings align with former findings from the IPISTOSS study, where iSSC showed a reduction in symptoms of depression in mothers and anxiety in fathers one week after birth.31 We can speculate that the effect on parents´ wellbeing enabled their capacity of parent-infant interaction, in favour of early bonding.

We did not correct for SSC time spent after the intervention for cortisol as explained above in your first question (Given the limited number of hypothesis-driven comparisons, we reported unadjusted p-values....)

VERSION 2 – REVIEW

**REVIEWER NAME**	Niermeyer, Susan
**REVIEWER AFFILIATION**	University of Colorado Denver School of Medicine, Pediatrics
**REVIEWER CONFLICT OF INTEREST**	No Conflict of Interest

**AI DISCLOSURE**	No, I have not used AI
**DATE REVIEW RETURNED**	07-Jan-2026

**GENERAL COMMENTS**	The authors have carefully addressed the reviewers' comments, and the manuscript has been improved by the details added on methods and analysis.<br><br>The expanded discussion of the difference in total amount of skin-to-skin care between the groups adds practical and important information that aids in interpreting the results.<br><br>There are a few minor issues introduced in the revisions:<br>p. 12, line 55-56 "in between" introduces a second preposition<br>p. 15, line 22-23 the introductory phrase seems incomplete, but it could be modified to "Although exploratory in nature, the analyses of infant and mother's……"<br>p. 19, line 3-4 "approuval" should be "approval"

**REVIEWER NAME**	**Dalrymple, Hannah**
**REVIEWER AFFILIATION**	The Children's Hospital at Westmead, Grace NICU
**REVIEWER CONFLICT OF INTEREST**	No Conflict of Interest
**AI DISCLOSURE**	No, I have not used AI
**DATE REVIEW RETURNED**	07-Jan-2026

**GENERAL COMMENTS**	Happy with amendments.

VERSION 2 – AUTHOR RESPONSE

We have adressed the comments to author from Reviewer 1 as following:

1) Reviewer 1: p. 12, line 55-56 "in between" introduces a second preposition:
Our modification: demonstrated a difference between iSSC ...

2) Reviewer 1: p. 15, line 22-23 the introductory phrase seems incomplete, but it could be modified to "Although exploratory in nature, the analyses of infant and mother's……"
Our modification: Although exploratory in nature, the analyses of infant and mother's in-moment coupling salivary cortisol levels revealed two contrasting trends of association between study groups.

3) Reviewer 1: p. 19, line 3-4 "approuval" should be "approval"
Our text: Ethics approval

4) We also changed the text slightly for more clarity in page 12 from "Preliminary analyses of GRS scores were made for Norwegian and Swedish samples separately and for the observation setting, home versus clinic with no significant differences" to " Preliminary analyses of GRS scores were made for country (Norway versus Sweden) and for the observation setting (home versus clinic), with no significant differences in scores between countries or settings".
